# Variable but consistent pattern of Meningioma 1 gene (*MN1*) expression in different genetic subsets of acute myelogenous leukaemia and its potential use as a marker for minimal residual disease detection

**DOI:** 10.18632/oncotarget.12269

**Published:** 2016-09-27

**Authors:** Sonia Carturan, Jessica Petiti, Valentina Rosso, Chiara Calabrese, Elisabetta Signorino, Giada Bot-Sartor, Paolo Nicoli, Daniela Gallo, Enrico Bracco, Alessandro Morotti, Cristina Panuzzo, Enrico Gottardi, Francesco Frassoni, Giuseppe Saglio, Daniela Cilloni

**Affiliations:** ^1^ Department of Clinical and Biological Sciences, University of Turin, Turin, Italy; ^2^ Department of Oncology, University of Turin, Turin, Italy; ^3^ Department of Pediatric Hemato-Oncology and Stem Cell and Cellular Therapy Laboratory, Institute G. Gaslini, Genova, Italy

**Keywords:** minimal residual disease, meningioma 1 gene, acute leukemias, molecular marker

## Abstract

Meningioma 1 (*MN1*) gene overexpression has been reported in acute myeloid leukaemia (AML) patients and identified as a negative prognostic factor. In order to characterize patients presenting gene overexpression and to verify if *MN1* transcript could be a useful marker for minimal residual disease detection, *MN1* was quantified in 136 AML patients with different cytogenetic risk and in 50 normal controls. In 20 patients bearing a fusion gene transcript suitable for minimal residual disease quantitative assessment and in 8 patients with *NPM1* mutation, we performed a simultaneous analysis of *MN1* and the fusion-gene transcript or *NPM1* mutation during follow-up. Sequential *MN1* and *WT1* analysis was also performed in 13 AML patients lacking other molecular markers. The data obtained show that normal cells consistently express low levels of *MN1* transcript. In contrast, high levels of *MN1* expression are present in 47% of patients with normal karyotype and in all cases with inv(16). *MN1* levels during follow-up were found to follow the pattern of other molecular markers (fusion gene transcripts, *NPM1* and *WT1*). Increased *MN1* expression in the BM during follow up was always found to be predictive of an impending hematological relapse.

## INTRODUCTION

The assessment of minimal residual disease (MRD) has currently become a necessary strategy to better address treatment intensity in acute leukemias [1]. The detection of MRD by RT-PCR is limited to those patients characterized by genetic markers. The latter include fusion genes derived from chromosome translocations, such as *PML-RAR*α *AML1* and *CBF-MYH11^2^* or mutations, for example nucleophosmin (*NPM1*), [1, 3] which has been validated as a sensitive marker of MRD detection. More recently, studies of next generation sequencing (NGS) allowed to enlarge the spectrum of genetic abnormalities by discovering new mutations and aberrations [4] Basing on these studies, other genetic markers are under investigation, including IDH1 and IDH2 mutations which occur in less than 10% of the patients [5].

Other genes found overexpressed in AML have been validated for MRD detection in many clinical settings. Among them, one of the most exploited is the Wilms tumor gene (*WT1*) [6–8].

Studies of NGS clearly showed that in AML at diagnosis there is a founding clone which prevails and several small subclones, characterized by different mutations, which are often undetectable by Sanger sequencing. These subclones can be selected by chemotherapy or by molecular targeted therapies and expand over time thus generating chemoresistance or relapse [4, 9].

Many studies suggest that *WT1*, although not associated with a specific leukemic clone, is very sensitive in the detection of the persistence or of the reappearance of the disease [8] The fact that its expression is not related to specific genetic alterations allows *WT1* to monitor the kinetic of the leukemic cells. Since *WT1* is not overexpressed only in AML but in other hematological malignancies including myelodysplastic syndromes [10] and myeloproliferative disorders, [11] it could be considered a “universal marker” of clonal hematopoiesis. Despite the fact that the majority of AML at diagnosis overexpresses *WT1*, in about 20-30% of AML the gene is not significantly overexpressed [8]. We therefore explored the possibility of additional molecular markers to monitor the disease.

The meningioma 1 gene (*MN1*), located on chromosome 22q11, was cloned from a patient affected by meningioma characterized by the translocation t(4;22) (p16;q11) [12]. Additional studies identified the fusion between *TEL* and *MN1* genes in AML patients with translocations t(12;22) (p13;q11) [13]. This genetic alteration, although very rare, represents a relevant prognostic factor with a negative impact on survival. Despite the role of this fusion transcript, it was shown that *MN1* overexpression represents a negative prognostic factor in terms of disease free survival [14].

Heuser and colleagues [14] investigated the significance of *MN1* expression in a uniformly treated cohort of adult AML patients with normal karyotype. In this study the prognostic relevance of *MN1* was compared to other prognostic factors such as *FLT3* internal tandem duplication (ITD), *MLL* and *NPM1* mutations. This study suggests that *MN1* overexpression is an independent prognostic marker in AML with normal karyotype and it is associated with shorter relapse free survival (RFS) and shorter overall survival (OS) [14].

The two main objects of the present study were the identification and characterization of the subset of patients showing *MN1* overexpression and the validation of *MN1* as a marker for MRD detection.

## RESULTS

### MN1 expression in AML patients at diagnosis

**Figure 1:**
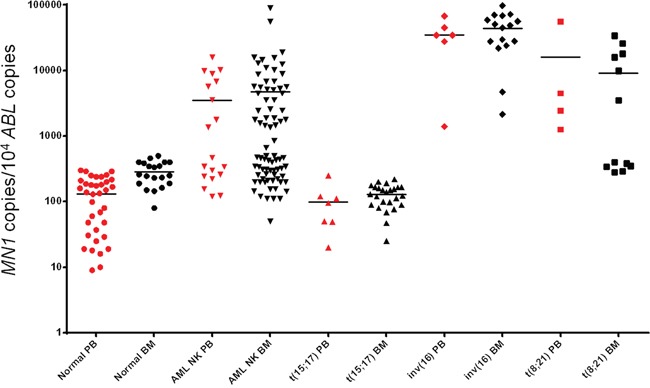
MN1 expression in PB (red dots) and BM (black dots) in samples from healthy volunteers, AML patients with normal karyotype, APL with t(15;17), AML with inv(16) and AML with t(8;21) chromosomal abnormalities The transcript amount is expressed as *MN1* copies/10^4^ *ABL* copies.

The expression levels of *MN1* transcript in normal controls and in leukemia samples at diagnosis are summarized in Table 1, 2 and Figure 1. The *MN1* levels were very low in normal samples: the mean copy number of *MN1*/10^4^ *ABL* copies is 130±94 (median 136; range 9-300) in peripheral blood (PB) and 285 ±117 in BM (median 254, range 80-500).

**Table 1:** *MN1* expression in normal and AML samples. BM= bone marrow, PB= peripheral blood NV= not valuable, SD= standard deviation, CTRL= healthy control

	Cytogenetic group	Type of samples	No. of samples tested	No. and percentage of patients with MN1 overexpression	*MN1 copies/10^4^ ABL copies*		
					Mean value ± SD	Median value	range
**CTRL**		BM	20		285±117	254	80-500
		PB	30		130±94	136	9-300
		CD34+	6		223±56	215	149-300
	**TOTAL**		**56**				
**AML**	normal karyotype	BM	79	37 (47%)	9766±16590	5136	852-90230
		PB	19	9 (47%)	7125±4663	6780	1367-15900
	t(15;17)	BM	25	0 (0%)	129±49	130	25-219
		PB	7	0 (0%)	99±75	95	20-250
	inv(16)	BM	16	16 (100%)	44270±26285	46950	2149-98000
		PB	6	6 (100%)	35200±21771	34500	1400-67999
	t(8;21)	BM	12	6 (50%)	17848±10925	16950	3500-34000
		PB	4	4 (100%)	16052±26665	3475	1260-56000
	complex K	BM	1	1 (100%)	21080	NV	NV
	t(9;22)	BM	1	1(100%)	9860	NV	NV
	trysomy 9	BM	1	1(100%)	8770	NV	NV
	5q-	BM	1	1(100%)	45935	NV	NV
	**TOTAL**		**172**				

**Table 2:** Clinical characteristics of the patients enrolled in the study. Treatment, indicated as A, B, C and D, is described in the “materials and methods” section

UPN	age	sex	cytogenetic	*NPM1*	*FLT3*	treatment	*MN1* copies/10000 ABL copies in BM	*WT1* copies/10000 ABL copies in BM	*NPM1* copies/10000 ABL copies
1	22	M	NK	y	N	A	199	156	10230
2	36	M	NK	y	Y	A	15900	3688	1860
3	66	F	NK	y	Y	B	199	270	6780
4	70	M	NK	y	N	B	111	189	7620
5	38	F	NK	y	N	A	145	340	NA
6	44	F	NK	y	N	A	199	1560	11831
7	49	F	NK	y	N	A	111	223	4210
8	57	F	NK	y	N	A	322	1700	NA
9	72	M	NK	y	N	B	444	568	NA
10	61	M	NK	y	Y	C	430	13400	3280
11	19	F	NK	y	N	A	90230	13200	8800
12	28	M	NK	y	N	A	10240	3400	NA
13	32	M	NK	y	Y	A	50	450	12555
14	45	M	NK	y	N	A	3570	22	NA
15	31	M	NK	y	Y	A	56000	3240	18340
16	60	F	NK	y	N	A	11100	390	2102
17	66	M	NK	y	Y	B	14637	12	NA
18	73	F	NK	y	N	B	265	590	NA
19	74	F	NK	y	N	B	300	200	9090
20	29	F	NK	y	N	A	1453	37	15910
21	18	M	NK	y	Y	A	111	11	NA
22	26	M	NK	y	N	A	2450	2350	2230
23	29	F	NK	y	N	A	4500	8090	NA
24	38	F	NK	y	N	A	5080	9030	NA
25	54	M	NK	y	N	A	344	88	13905
26	58	M	NK	y	N	A	1460	9000	NA
27	73	M	NK	y	N	B	2466	45	17600
28	70	F	NK	y	N	B	8799	23	NA
29	48	F	NK	y	N	A	666	1900	NA
30	68	F	NK	y	Y	B	156	340	1190
31	44	M	NK	y	N	A	468	1200	NA
32	41	M	NK	y	N	A	2566	18	220
33	50	F	NK	y	N	A	243	2510	NA
34	74	M	NK	y	N	B	222	2560	NA
35	73	F	NK	y	N	B	1890	40	80279
36	30	M	NK	y	N	A	1570	62	NA
37	49	F	NK	y	N	A	5555	5200	NA
38	45	M	NK	y	N	A	400	250	7700
39	27	M	NK	y	Y	A	345	660	NA
40	69	F	NK	y	N	B	6870	66	NA
41	22	F	NK	y	N	A	1790	21900	2200
42	30	M	NK	y	N	A	5666	10300	NA
43	68	M	NK	y	N	B	3573	10	NA
44	65	M	NK	y	N	B	1888	23200	14200
45	61	F	NK	y	N	A	12500	8700	NA
46	45	M	NK	N	N	A	120	7800	
47	31	M	NK	N	N	A	258	2140	
48	30	F	NK	N	Y	A	340	220	
49	48	F	NK	N	N	A	422	900	
50	44	F	NK	N	N	A	254	8560	
51	48	F	NK	N	N	C	18890	22	
52	31	F	NK	N	D835	A	299	334	
53	64	M	NK	N	N	A	154	14500	
54	63	M	NK	N	N	C	197	2230	
55	20	F	NK	N	N	A	15730	13200	
56	46	M	NK	N	N	A	13000	78	
57	66	F	NK	N	N	B	312	223	
58	33	M	NK	N	Y	A	311	1880	
59	47	M	NK	N	N	A	4500	34	
60	74	F	NK	N	N	B	500	890	
61	60	F	NK	N	N	C	211	228	
62	61	M	NK	N	N	A	466	2460	
63	70	M	NK	N	N	B	444	2184	
64	22	M	NK	N	N	A	499	3340	
65	50	M	NK	N	N	A	476	5510	
66	49	F	NK	N	N	C	311	1990	
67	32	F	NK	N	N	A	6780	28	
68	72	F	NK	N	N	B	8900	88	
69	44	F	NK	N	N	A	222	750	
70	70	M	NK	N	D835	B	143	145	
71	28	M	NK	N	N	A	5136	2200	
72	36	F	NK	N	N	A	5305	5780	
73	49	M	NK	N	N	A	852	31	
74	51	F	NK	N	N	A	1674	8800	
75	70	M	NK	N	N	B	1367	3280	
76	66	M	NK	N	D835	B	1790	1120	
77	42	F	NK	N	Y	A	200	676	
78	26	F	NK	N	N	A	210	2250	
79	19	F	NK	N	N	A	5680	34	
80	56	F	t(15;17)	N	N	D	192	13450	
81	66	F	t(15;17)	N	N	D	165	23410	
82	34	M	t(15;17)	N	N	D	177	7850	
83	51	M	t(15;17)	N	N	D	219	8400	
84	58	M	t(15;17)	N	N	D	200	29800	
85	38	M	t(15;17)	N	N	D	166	78400	
86	44	F	t(15;17)	N	Y	D	130	17320	
87	50	M	t(15;17)	N	N	D	167	54700	
88	39	F	t(15;17)	N	N	D	155	3300	
89	51	F	t(15;17)	N	N	D	150	7120	
90	49	M	t(15;17)	N	N	D	90	2650	
91	48	M	t(15;17)	N	Y	D	25	11070	
92	44	F	t(15;17)	N	N	D	122	62190	
93	55	F	t(15;17)	N	N	D	160	9240	
94	60	F	t(15;17)	N	N	D	150	8840	
95	44	M	t(15;17)	N	N	D	140	3780	
96	49	F	t(15;17)	N	N	D	113	14200	
97	52	M	t(15;17)	N	N	D	80	5891	
98	56	F	t(15;17)	N	N	D	69	830	
99	41	F	t(15;17)	N	N	D	47	41690	
100	65	M	t(15;17)	N	N	D	76	27260	
101	59	M	t(15;17)	N	N	D	98	16980	
102	55	M	t(15;17)	N	Y	D	89	27120	
103	45	M	t(15;17)	N	N	D	100	20050	
104	62	F	t(15;17)	N	N	D	120	15380	
105	49	F	t(8;21)	N	N	A	34000	2180	12500
106	21	F	t(8;21)	N	N	A	3500	1650	6580
107	68	F	t(8;21)	N	N	B	9900	880	2490
108	35	M	t(8;21)	N	N	A	18000	1530	57810
109	51	M	t(8;21)	N	N	A	25788	14300	2250
110	66	F	t(8;21)	N	N	B	15900	4300	1570
111	32	M	t(8;21)	N	N	A	400	910	22500
112	56	F	t(8;21)	N	N	A	340	22800	4780
113	41	M	t(8;21)	N	Y	A	290	18300	7630
114	34	M	t(8;21)	N	N	A	350	5600	10300
115	48	M	t(8;21)	N	N	A	280	3490	8700
116	68	F	t(8;21)	N	N	B	380	19200	22800
117	29	F	inv(16)	N	N	A	21935	750	21192
118	59	M	inv(16)	N	N	A	24128	3190	9027
119	41	F	inv(16)	N	N	A	72022	1840	13580
120	42	F	inv(16)	N	N	A	98000	2600	8920
121	49	F	inv(16)	N	N	A	50900	2530	6510
122	22	M	inv(16)	N	N	A	59000	4160	62800
123	46	F	inv(16)	N	N	A	70280	3140	11519
124	51	M	inv(16)	N	N	A	68900	1020	37911
125	69	M	inv(16)	N	N	B	28000	1010	80134
126	39	M	inv(16)	N	N	A	45000	47620	10100
127	29	F	inv(16)	N	N	A	27900	5610	10180
128	60	F	inv(16)	N	N	A	4700	5990	12816
129	66	F	inv(16)	N	N	B	29804	6230	11280
130	61	M	inv(16)	N	N	A	56700	980	29880
131	46	F	inv(16)	N	N	A	48900	4700	10065
132	48	M	inv(16)	N	N	A	2149	390	NA
133	30	F	complex K	N	N	C	21080	36880	
134	47	M	t(9;22)	N	N	A	9860	21370	
135	60	M	46XY;+9	N	N	C	8770	28600	
136	21	F	46XX;-5q	N	N	A	45935	990	

Similarly, low levels of expression were detected in normal CD34+ cells obtained from healthy volunteers: mean *MN1* copies/10^4^ *ABL* copies 223±56 (median 215 copies/10^4^ *ABL* copies, range 149-300).

Conversely, as shown in Table 1 and 2, 47% of the samples collected at diagnosis from AML patients characterized by a normal karyotype showed abnormal expression of the *MN1* gene. In this subset of patients, the mean value of expression evaluated for 37 out of 79 BM samples showed a transcript amount above the upper limit of normal controls is 9707±16590 copies/10^4^ *ABL* copies (median 5136, range 852-90230). Interestingly, as shown in Figure 1, NK AML and CBF AML seem to segregate in two groups, one with normal values (below 500 *MN1* copies/10^4^ ABL copies for BM and 300 *MN1* copies for PB), the second with *MN1* values above 1000 copies. This raises the possibility of a “gray zone” between positivity and negativity. At present we cannot establish the prognostic significance of *MN1* with values falling in that range.

In accordance, 9 out of 19 PB samples presented abnormal expression with a mean value of 7125 ± 4663 (median 6780, range 1367-15900). All samples carrying the fusion transcript *CBβ-MYH11* expressed a significantly higher amount of *MN1* transcript. The mean copy number is 44270±26285 (median 46950, range 2149-98000) in BM and 35200±21771 (median 34500, range 1400-67999) in PB. These values are significantly higher as compared to controls (p<0.0001 in both BM and PB). Fifty % of the samples characterized by the fusion gene *RUNX1-AML1* abnormally expressed *MN1.* The mean value of *MN1* copies calculated for BM cells with *MN1* expression above the upper limit established by normal samples was 17848±10925 (median 16950, range 3500-34000). All four PB samples tested presented high *MN1* values with a mean copy number of 16052±26665 (median 3475, range 1260-56000). Additionally, four AML patients with sporadic abnormalities such a t(9;22), trisomy 9, 5q-, and complex karyotype were included. All expressed abnormal *MN1* transcript values (data shown in Table 1). Finally, the Acute Promyelocytic Leukaemia (APL) samples expressed *MN1* values comparable to those of healthy subjects in both BM (p= 0.4) and PB (p=0.08).

**Figure 2:**
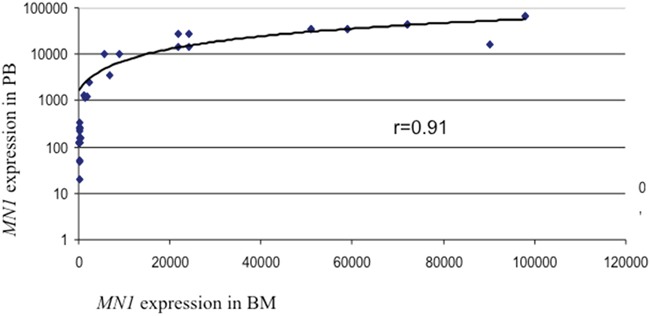
Correlation between MN1 expression in PB and BM The transcript amount is expressed as *MN1* copies/10^4^ *ABL* copies.

Interestingly, the paired analysis of 47 PB and BM samples collected from the same cohort of patients allowed us to establish a remarkable correlation between *MN1* expression in PB and BM. Regression analysis provided an r value of 0.91 (Figure 2).

Stratification of patients according to the presence of *FLT3* mutation or internal tandem duplication (ITD) demonstrated no significant association between the two abnormalities. *MN1* was overexpressed in 35% of patients with *FLT3* ITD, 33% of patients with the D835 mutation and 50% of those with wild type *FTL3*.

Finally, in contrast to previously published data, we were unable to find any significant correlation between *EVI-1* and *MN1* expression (r= 0.06) or between *MN1* and *NPM1* mutation (r=0.2).

### MN1 as a target for MRD detection

**Figure 3:**
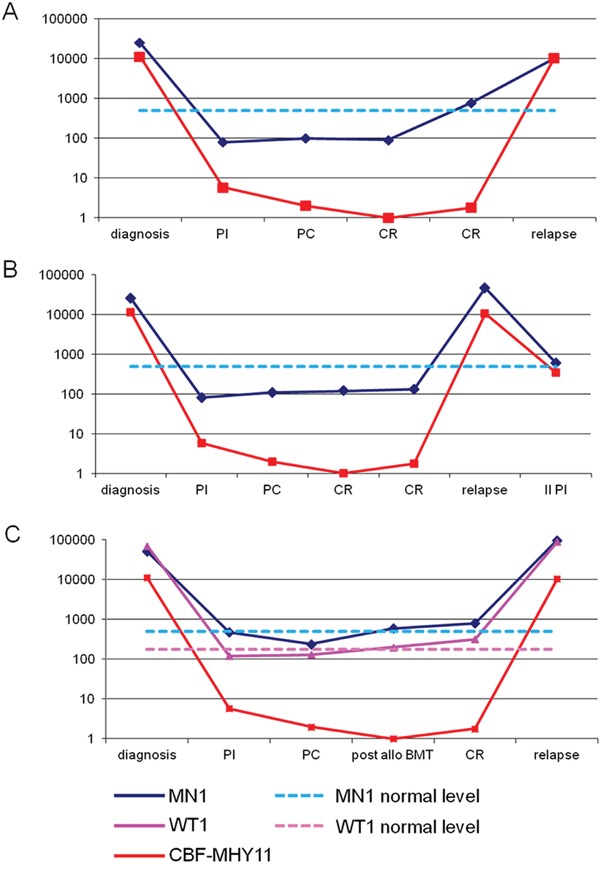
**Panel A and B:** *MN1* transcript expressed as number of copies/10^4^ *ABL* copies (blue line) and *CBFβ-MYH11* transcript expressed as copy number/10^4^ *ABL* copies (red line) at diagnosis and during follow-up in two patients with inv(16) who relapsed. *MN1* increased above the upper normal limit three months before relapse in patient represented in panel A. **Panel C**: *MN1* transcript (blue line), *CBFβ-MYH11*transcript (red line) and *WT1* transcript (pink line) at diagnosis and during follow-up of a patient with an inv(16) alteration who relapsed after two cycles of chemotherapy and allogeneic bone marrow transplantation (allo BMT). *MN1* and *WT1* increased above the upper normal limit four months before haematological relapse. PI= post induction, PC= post consolidation, CR= complete remission.

To assess the significance of *MN1* expression as a marker for MRD detection in AML, the *MN1* transcript amount was quantified during follow-up in 20 AML patients characterized by the presence of specific fusion-gene transcripts (15 *CBFβ*-*MYH11* and 5 *RUNX1-AML1*), in 8 patients with NPM1 mutation and in and 13 AML patients (including 3 resistant cases) lacking additional molecular markers but monitored by making use of *WT1* quantitative assessment, which we have previously demonstrated to strictly parallel fusion gene transcript behavior [6]. In all cases characterized by the presence of a fusion-gene transcript, the longitudinal pattern of *MN1* expression was always found to parallel that of the fusion gene. (Table 3) Three representative AML cases are illustrated in Figure 3. In the inv(16) AML subgroup, the patient who remained in continuous complete remission (CCR) constantly showed *MN1* values within the normal range (Table 3), whereas the five patients who ultimately relapsed showed a progressive raising of *MN1* levels above the normal range during hematological remission (Table 3 and Figure 3). In the cases illustrated in Figure 3 panel A and C, *MN1* values were found above the normal range in concomitance with quite stable values of *CBFβ-MYH11* transcript in BM samples taken 3 and 4 months, respectively, before hematological relapse while the patients were still in hematological remission.

**Table 3:** Simultaneous evaluation of the expression of *MN1* and fusion gene transcript (*CBF-MYH11* or *RUNX1-AML1)* or *NMP1* mutation during follow up in patients with AML

**Pt**	**Target gene**	**Diagnosis**	**Post Induction**	**CR Post consolidation I**	**CR pst Post consolidation II**	**follow-up**	**Relapse**
1	MN1	21935	210	190	192		
	CBF-MYH11	21192	167	12	12		
2	MN1	24128	87	90	100		
	CBF-MYH11	9027	18	9	11		
3	MN1	72022	312	290	97		
	CBF-MYH11	13580	212	21	8		
4	MN1	98000	95	88	90		
	CBF-MYH11	8920	15	21	12		
5	MN1	50900	320	190	261	190	
	CBF-MYH11	6510	134	67	52	30	
6	MN1	59000	110	80	90		
	CBF-MYH11	62800	180	200	80		
7	MN1	70280	88	113	180	883	3714
	CBF-MYH11	11519	340	391	120	312	19711
8	MN1	68900	37	41	22		
	CBF-MYH11	37911	412	91	11		
9	MN1	28000	512	193	114	121	
	CBF-MYH11	80134	670	120	69	21	
10	MN1	45000	820	632	880	920	53490
	CBF-MYH11	10100	8	4	1	3	9980
11	MN1	27900	91	102	100	920	10142
	CBF-MYH11	10180	8	6	1	5	10090
12	MN1	4700	66	61	45		
	CBF-MYH11	12816	34	12	4		
13	MN1	29804	97	112	118	121	32560
	CBF-MYH11	11280	8	4	1	4	10120
14	MN1	56700	180	134	153		
	CBF-MYH11	29880	12	15	12		
15	MN1	48900	812	410	880	970	10103
	CBF-MYH11	10065	8	6	1	3	10012
**Pt**	**Target gene**	**Diagnosis**	**Post Induction**	**CR Post consolidation I**	**CR pst Post consolidation II**	**follow-up**	**Relapse**
16	MN1	34000	211	170	145	161	
	RUNX1-AML1	97170	4720	266	27	64	
17	MN1	3500	118	111	131	880	18670
	RUNX1-AML1	9027	19	7	5	12	8650
18	MN1	9900	407	112	109		
	RUNX1-AML1	11830	229	15	2		
19	MN1	18000	110	141	390	1020	28910
	RUNX1-AML1	9902	51	4	3	13	12500
20	MN1	25788	99	81	17		
	RUNX1-AML1	2250	22	12	8		
**Pt**	**Target gene**	**Diagnosis**	**Post Induction**	**CR Post consolidation I**	**CR pst Post consolidation II**	**follow-up**	**Relapse**
21	MN1	15900	113	103	190	1020	24500
	NPM1	1860	21	11	6	54	2830
22	MN1	90230	90	81	52		
	NPM1	8800	21	2	1		
23	MN1	56000	2200	860	920	4290	82104
	NPM1	18340	25	28	31	72	11454
24	MN1	1453	105	99	92		
	NPM1	15910	320	76	34		
25	MN1	2466	990	800	720	1660	6792
	NPM1	17600	120	80	31	60	6505
26	MN1	2566	62	71	34		
	NPM1	220	12	2	0		
27	MN1	1890	63	61	12		
	NPM1	80279	12	1	1		
28	MN1	1790	41	44	13		
	NPM1	2200	5	0	0		
**Resistant pt**	**Target genes**	**Diagnosis**	**Post Induction**				
29	MN1	38910	25200				
	WT1	8450	9240				
30	MN1	82100	72120				
	WT1	7220	6123				
31	MN1	42193	63408				
	WT1	9920	13450				

Similarly, in the t(8;21) group, three patients who were in CCR never showed levels of *MN1* transcript above the normal range (Table 3), whereas in the two patients who relapsed, increased *MN1 l*evels were detectable 1 and 2 months respectively before the evidence of relapse.

In addition in 8 patients with *NPM1* mutation the quantitative analysis of *MN1* and *NPM1* shows a concordance between the two markers with a progressive increase of both before relapse and normal values of *MN1* and negative *NPM1* during remission.

**Figure 4:**
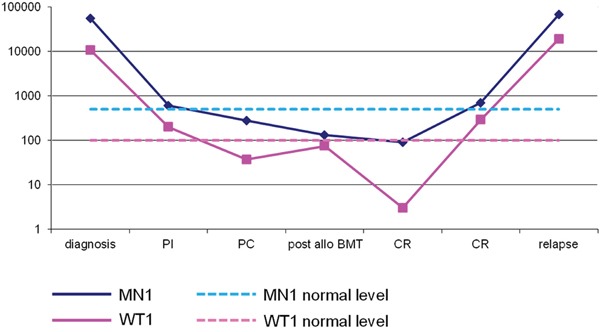
MN1 transcript (blue line) and WT1 transcript (pink line) expressed as number of copies/104 ABL copies at diagnosis and during follow-up of a patient with normal karyotype who obtained remission after chemotherapy and allogeneic bone marrow transplant and relapsed six months after transplant In this patient, both markers, *WT1* and *MN1*, returned to normal range and increased three months before relapse.

Finally, all patients were also monitored using *WT1* quantitative assessment. As shown in Figure 3 panel C, and as already demonstrated in our previous study and in many studies from the literature, [15] *WT1* strictly paralleled the behaviour of the fusion transcripts. Furthermore, we found that *MN1* strictly paralleled *WT1* in patients without any fusion gene (Figure 4) and in patients with rearrangements in the core binding factor (Figure 3C). Figure 4 shows the two molecular markers used during follow up of a patient who obtained a remission after two courses of chemotherapy and allogeneic bone marrow transplant. In this patient, both markers, *WT1* and *MN1*, returned to normal range and both increased three months before relapse.

Although the presented examples show a good degree of concordance in the curves representing *MN1* and fusion genes, they are not completely parallel. In some cases it seems that *MN1* expression, similarly to *WT1*, is cleared more rapidly than fusion gene transcripts during induction of remission, but it also seems that its elevation is more indicative than the fusion gene transcript in predicting relapse (see Figure 3 panel A and C). At the moment this cannot be easily explained due to our lack of knowledge about the kinetics of *MN1* expression in AML.

## DISCUSSION

The negative impact of the persistence of minimal residual disease after chemotherapy or before bone marrow transplantation in acute myeloid leukemia patients is well established [1]. RT-PCR is among the most sensitive methods used for the quantification of the MRD but it requires the presence of genetic markers in the leukemic clone including fusion transcripts or mutations [2, 3].

The disease monitoring became even more cumbersome with the genomic characterization of AML which elegantly demonstrated that, al least at onset, the acute leukemia is mainly constituted by a founding clone with a variable number of mutations and by additional subclones, nearly undetectable, carrying mutations different from the founding clone [4, 9].

These subclones might eventually be selected during chemotherapy and expand during the course of the disease [4, 9]. Considering the dynamic of the leukemic clones it should not be unpopular to suggest the use of a marker not specifically related to a clone but able to identify the presence of leukemic cells independently from their genetic lesions and their phenotype.

In addition, we must consider that in many laboratories NGS technology is not yet available and RQ-PCR targeting all the identified mutations is time consuming and expensive. The advantage of using a single “universal marker” with high sensitivity and specificity allow to better monitor the disease during therapy and in the remission phase.

Interestingly, the association of *MN1* with myeloid malignancy goes beyond *MN1* involvement in rare translocations such as t(12;22), as the gene is overexpressed in a significant percentage of AML patients. These has been already demonstrated in literature in some patients characterized by overexpression of the transcription factor ectropic viral integration 1 site (*EVI1*) [16] and in some adult AML patients without karyotypic abnormalities. In the latter case, overexpression of *MN1* was associated with a worse prognosis and shorter survival rate [14]. Despite the possibility that *MN1* could represent an independent prognostic factor for AML patients, particularly for those with a normal karyotype, there are few data regarding the expression of *MN1* in normal hematopoietic cells and in different subtypes of AML. Furthermore, there is currently no evidence that *MN1* could represent a suitable marker for minimal residual disease detection. Using a real time quantitative PCR approach, we show that *MN1* expression is detectable in all normal bone marrow and peripheral blood samples and CD34 positive cells collected from healthy subjects, although we were able to estimate that normal subjects expressed very low *MN1* levels. In contrast, a significant number of patients are characterized by high *MN1* transcript amount and, in several cases, the expression is at least 2 or 3 logs higher than controls. It appears that overexpression of *MN1* is mainly associated with the inv16 chromosomal abnormality and with a normal karyotype, whereas in t(15;17) APL the values are consistently comparable to controls. In contrast, we were unable to find any significant correlation between *FLT3* ITD or mutation or *EVI-1* overexpression. Moreover, the finding that CD34-positive cells express low levels of *MN1* transcript supports the notion that increased levels of *MN1* expression are indeed specific of leukemic blasts and not a simple consequence of the degree of differentiation. Since a significant percentage of AML shows consistently increased *MN1* expression levels, this could represent a candidate marker suitable to discriminate between normal and leukemic hematopoiesis and useful to establish the presence, persistence or reappearance of leukemic clone. In particular, our data show that 45% of AML cases lacking other molecular markers suitable for MRD monitoring express at diagnosis *MN1* transcript values above the normal range established by healthy subjects. In this subset of patients, *MN1* may represent a reliable marker for MRD detection. So far no data are available concerning the clinical significance of detection of *MN1* expression by RT-PCR for monitoring patients with acute leukemia during follow-up. The data presented in this paper show that an accurate quantitative assessment of *MN1* transcript amount allows to clearly distinguish between normal and abnormal expression levels of *MN1* and, as for *WT1*, can overcome the problem represented by the minimal amount of gene expression found in normal hematopoietic progenitors. The simultaneous quantitative assessment of the *MN1* transcript and of the specific fusion gene or *NPM1* mutation showed a good parallelism between the behaviour of the two markers. Indeed, minor discrepancies at low levels of expression of the two markers were observed. In particular, the decrease in *MN1* expression seems to be particularly rapid compared to the fusion gene transcript during the induction of remission and its elevation before relapse is more rapid and therefore it is probably more sensitive in predicting relapse. Therefore, even though the degree of sensitivity for MRD detection by analysis of *MN1* expression remains to be established, the results obtained show that an increase in *MN1* expression above normal levels can be of prognostic significance in predicting relapse during follow-up of AML patients. Although the *MN1* gene requires validation as a marker for minimal residual disease in future prospective studies, it seems to be a promising marker for this purpose and further studies should be encouraged.

## MATERIALS AND METHODS

After informed consent, 136 acute myeloid leukaemia patients and 50 healthy volunteers were included in the study. 136 bone marrow samples (BM) and 36 paired peripheral blood samples (PB) were collected from 136 AML patients at diagnosis. In addition, 41 patients were studied during follow-up. The median age was 48 years (range 18-74). All cases were classified according to FAB criteria, characterized at the cytogenetic level by conventional karyotyping and screened by RT-PCR for the presence of the most frequent fusion transcripts, as previously described.^2^ *NPM1* mutations ^3^ and *FLT3* ITD or D835 mutations were screened. *WT1* quantitative assessment is available for all samples included in the study and, furthermore, in 40 out of 136 BM samples *EVI-1* quantitative assessment was also performed. The FAB distribution was as follows: FAB M0=21, FAB M1= 22, FAB M2=26, FAB M3= 25, FAB M4= 24, FAB M5=16, FAB M6=2. Patients younger than 60 years were treated following standard protocols established by the GIMEMA Cooperative Group for the treatment of adult patients with acute myeloid leukemia which included: Induction treatment with a 3-drug regimen: Daunorubicine (DNR) 50 mg/sqm/day on days 1, 3 and 5; Cytosine-Arabinoside (ARA-C) 100 mg/sqm/day on days 1 to 10; Etoposide 100 mg/sqm/day on days 1 to 5; to be repeated in case of partial remission (PR). Consolidation therapy with DNR 50 mg/sqm/day on days 4 to 6 and intermediate-doses ARA-C (500 mg/sqm/12 h on days 1 to 6) for patients achieving complete remission (CR) after either the first or the second induction cycle.

Additional consolidation treatments with high dose ARA-C were used followed, in high risk patients by allogeneic stem cell transplantation. (This regimen is indicated as treatment A in Table 2) Elderly or unfit patients were treated with two cycles of daunorubicin 45 mg/sqm/day on days 1, 3 plus ARA-C 100 mg/sqm/day on days 1 to 7 followed, in same cases, by autologous stem cell transplantation. (This regimen is indicated as treatment B in Table 2)

Refractory or secondary AML were treated following the Mito-FLAG scheme (Fludarabine 30 mg/sqm day 1-5, ARA-C 2000 mg/sqm day 1-5, mitoxantrone 7 mg/sqm day 1,3,5 and G-CSF 5 μg/kg from day −1) and consolidated as described above. (This regimen is indicated as treatment C in Table 2). Finally Acute promyelocytic leukemia were treated with anthracycline-based risk-adapted chemotherapy plus all-trans retinoic acid (ATRA) [17]. (This regimen is indicated as treatment D in Table 2)

Complete remission was defined according to standard criteria. Finally 30 PB and 20 BM and 6 CD34+ enriched peripheral blood stem cell samples collected from healthy volunteers were included as normal control.

### Real time quantitative RT-PCR (RQ-PCR) analysis of MN1 and WT1

Total RNA was extracted using TRI Reagent solution (Ambion, Waltham, MA USA). Mononuclear cells were separated on a Ficoll–Hypaque density gradient. Total RNA was extracted by standard procedure. The RT (reverse transcription) step was performed as previously described [2, 8]. RQ-PCR reactions and fluorescence measurements were made on the ABI PRISM 7700 Sequence Detection System (PE Applied Byosystems, Foster City, USA).

Briefly, the RQ-PCR primers and probe for *MN1* detection were provided by ELItech, Turin, Italy

Primers and probe for *MN1* detection are:

5′ AGAAGGCCAAACCCCAGAACC-3′

5′ GATGGTGAGGCCTTGTTTGCA-3′

5′ Fam-ACAGCAAAGAAGCCCAC-MGBNFQ 3′

For *WT1* we followed the ELN standardized method reported [8].

The analysis was performed in triplicate and results showing a discrepancy >1 Ct in one of the wells were excluded and repeated. For quantitative assessment of *MN1* a calibration curve with a plasmid containing *MN1* target sequences was used (ELItech, Turin, Italy). The *MN1* values obtained by RQ-PCR were normalized with respect to the number of *ABL* transcripts and expressed as *MN1* copy number every 10^4^ copies of *ABL*. Quantitative assessment of *CBF*-*MYH11* and *RUNX1-AML1* transcripts was determined using primers and probes according to standardized procedures.^2^

### Real time quantitative RT-PCR (RQ-PCR) analysis of EVI-1

For *EVI-1* and *ABL* quantification, specific assays on demand kits of primers and probe (assay ID for *EVI-1* HS01118675_m1 and for ABL Hs00245445_m1 (Applied Byosystems, Foster City, USA) were used following the manufacturer's instructions.

All sample analysis was performed in triplicate and results showing a discrepancy >1 Ct in one of the wells were excluded and repeated.

*EVI-1* Ct obtained by RQ-PCR was normalized with respect to the Ct of *ABL* and calibrated with universal RNA (Stratagene, Santa Clara, California, USA) and finally expressed as 2^−ΔΔCt^.

### CD34-positive cells enrichment

CD34+ cells were enriched according to a magnetic cell sorting methodology (MACS; Miltenyi Biotec, Bergisch Gladbach, Germany). Briefly, mononuclear cells were labeled with a haptenized CD34 antibody (QBEND\10) that was magnetically labelled in a second step reaction with an anti-hapten antibody coupled to super paramagnetic microbeads. Labelled cells were then separated using a high gradient magnetic separator column placed in a strong magnetic field. The magnetically stained cells were retained in the column, and when the latter was removed from the magnetic field, CD34-positive cells were eluted. At the end of the procedure, CD34 positive cells represented more that 90% of the total as determined by flow cytometric analysis.

### Statistical analysis

*MN1* values obtained for different types of leukemia were compared using the Student's *t*-test.
